# Aneurism of the Middle Meningeal Artery—Fatal Operation

**Published:** 1829-01-01

**Authors:** 


### XXVIII.

### ANEURISM OF THE MIDDLE MENINGEAL ARTERY—FATAL OPERATION. By Dr. KRIMER.

The following melancholy case is recorded in a late number of Graef's Journal, by Dr. Krimer.

*Case.* A peasant, of strong and good constitution, had received a blow on the left temple two years ago, after which, an indolent tumour gradually formed on the part, attended with pain in the head. This tumour was hard, moveable, round in form, and about the size of a nut. The surgeon of the place where the peasant resided considered it as an encysted tumour, and Dr. K. being consulted, was of the same opinion, recommending its removal by the knife, care being taken to avoid wounding the temporal artery and muscle. The surgeon found that the supposed cyst was beneath the temporal muscle, which he divided in the direction of its fibres. The tumour, then, on being isolated, appeared to be connected to the periosteum by a kind of peduncle, the size of a goose-quill. This was separated by a stroke of the knife, when there instantly burst forth a tremendous, and ultimately fatal hæmorrhage. Dr. K. was summoned, but before he arrived the patient was dead.

The dissection was imperfect; but the disease was proved to be an aneurism of

---

the middle artery of the dura mater, which had made its way outwards, through the suture uniting the squamous portion of the temporal bone, to the corresponding portion of parietal bone. It appears that a considerable extravasation of blood had taken place in the interior of the skull, and to this, rather than the external hæmorrhage, the death of the patient is attributed. The case may form a lesson of caution to surgeons, when operating for tumours situated in the situation above-mentioned. The arteria meningea media was found to be the size of a man's finger, near the place where the aneurism emerged from between the bones.

